# Pancreatic stellate cells regulate blood vessel density in the stroma of pancreatic ductal adenocarcinoma

**DOI:** 10.1016/j.pan.2016.05.393

**Published:** 2016

**Authors:** Francesco Di Maggio, Prabhu Arumugam, Francesca R. Delvecchio, Silvia Batista, Tanguy Lechertier, Kairbaan Hodivala-Dilke, Hemant M. Kocher

**Affiliations:** a Centre for Tumour Biology, Barts Cancer Institute – a CRUK Centre of Excellence, Queen Mary University of London, London EC1M 6BQ, UK; b Barts and the London HPB Centre, The Royal London Hospital, Barts Health NHS Trust, London E1 1BB, UK

**Keywords:** Pancreatic stellate cells, Angiogenesis, Micro-environment, Juxta-tumoral, Panstromal

## Abstract

**Background/objectives:**

The vascular heterogeneity of pancreatic ductal adenocarcinoma (PDAC) has never been characterised. We analysed the heterogeneous vascular density of human PDAC along with its prognostic correlation.

**Methods:**

Tissue Microarrays of 87 patients with different pancreatico-biliary pathologies were analysed in an automated manner (Ariol™) after CD31 staining to assess vascular density in juxta-tumoral and panstromal compartments. *In vitro* and *ex vivo* assays were carried out to assess the role of PSC.

**Results:**

PDAC has a distinct vascular density and distribution of vessels compared to cholangiocarcinoma. The PDAC juxta-tumoral stroma was hypovascular and the normal adjacent rim was hypervascular compared to the panstromal compartment. These features adversely affected patient prognosis, suggesting a model for spatio-temporal PDAC evolution. Mice aortic rings and 3D organotypic cultures demonstrated pro- and anti-angiogenic signalling from activated PSC and cancer cells respectively. ATRA-induced quiescence suppressed the pro-angiogenic activity of PSC.

**Conclusion:**

Human PDAC has variable vascularity at microscopic level suggesting that novel stromal directed therapies would need to be determined by pathological characteristics.

## 1 Introduction

Pancreatic ductal adenocarcinoma (PDAC) is characterised by loss of acino-lobular architecture, progressive subversion of the stromal/epithelial ratio and extracellular matrix (ECM) deposition [1]. Pancreatic stellate cells (PSC) are the conductors of the microenvironmental disruption contributing to PDAC growth, early invasion and dismal prognosis [1], [2]. The hypovascular attribute of PDAC is routinely utilised in the clinic for its diagnosis by imaging such as CT scan [3]. The hypovascular nature and diffuse fibrosis is also noted at histological examination [4]. In fact, this aspect has been highlighted in experimental conditions to account for lack of therapeutic effect of chemo- and radio-therapy [4], [5], [6].

The desmoplastic stroma is a dynamic temporally and spatially distinct compartment of PDAC that is critically involved in tumour formation and progression and may influence the vascularity. Our recent studies have revealed a differential inflammatory infiltrate in the juxta-tumoral (<100 μm) and pan-stromal compartment in the human PDAC, which may be due to multitude of cytokines, chemokines and adhesion molecules produced by this desmoplastic stroma [7], [8], [9], [10]. We postulated that these differences between the stromal sub-compartments may also exist for vascular density. Here, we demonstrate that pancreatic stellate cells (PSC) are responsible for the changes in the observed tumoral and stromal sub-compartmental vascular density, which may be of prognostic relevance.

## 2 Materials and methods

### 2.1 Tissue microarray analyses

Tissue microarrays (TMAs) were constructed with pancreatic tissues from 87 HPB (Hepato-Pancreatico-Biliary) patients subjected to pancreatic resection (Supplementary Table 1) at Barts Health NHS Trust (City and East London Research Ethics Committee 07/0705/87) as described previously [7]. Regions of tumour, stroma, and normal pancreas were marked on H&E-stained slides of the donor tissue blocks, and three 1-mm cores of each region were sampled per patient using the Tissue Arrayer Minicore 3 (Alphelys, Plaisir, France). TMAs were stained with CD31 antibody (Rabbit anti Human ab28364 Abcam, Cambridge, MA) for the presence of vascular structures. Micro Vascular Density (MVD = number of vessels/mm^2^) and Total Vascular Area (TVA = area occupied by vascular structures as proportion of the total area expressed in/1000 or ‰) were considered as indices of vascularisation [11], [12], [13]. The Ariol™ imaging analysis system (Genetix, New Milton, England) was used to determine vascular indices. Briefly, software is trained by the user to distinguish and quantify positive cells by their colour, shape and size as described previously [7]. Cores were initially divided into “normal” (derived from patients) with other malignancies such as duodenal cancers or neuroendocrine tumours (pancreas more than 1 cm from tumour mass), “cancer” and “stroma” (the latter being cancer patients' tissues containing no epithelial component) for PDAC or cholangiocarcinoma.

PDAC-associated stroma was then divided into “juxta-tumoral” (≤100 μm from the epithelial cancer component) and “pan-stromal” [7]. The juxtatumoral compartment was marked using imaging callipers on all TMA cores. The panstromal vascular density was calculated by subtracting the tumoural and juxtatumoral area from each TMA core and then dividing the relevant number of vessels by it. Ariol™ software detects the surface area occupied by structures measured. Finally, microscopically healthy looking tissues (obtained from PDAC patients) surrounding tumours were also analysed (normal adjacent) and compared to the other groups. The median values of all analysed tumour cores for each patient within different TMA regions were used to determine the prognostic impact of all the indices.

### 2.2 Isolation of PS-1, human telomerase reverse transcriptase, immortalization of PS-1 cells

Using the outgrowth method pancreatic stellate cells were isolated from an unused donated human pancreas (donation for transplantation) by the UK Human Tissue Bank (Ethics approval; Trent MREC, 05/MRE04/82). The resulting cell strain, designated PS-1, was verified as being of stellate cell origin (grown in E4:F12 medium). PS-1 cells were immortalized by 24 h incubation with retroviruses containing cDNA encoding human telomerase reverse transcriptase (hTERT) derived from the AM12 packaging cell line (AM12-hTERT) with empty-vector transduced controls and selected with 1 g/ml puromycin. Immortalized cell telomerase activity was ascertained by the TRAP assay (Telomerase Repeat Amplification Protocol, Oncor, Inc.; manufacturer's instructions).

### 2.3 Cell cultures and organotypic cultures

PSC (Pancreatic Stellate Cells) and PCC (Pancreatic Cancer Cells) such as Capan1, Colo357 and AsPc1 were cultured in a standard manner as described before [10]. Human Umbilical Vein Endothelial Cells (HUVEC) hTERT (a kind gift from Prof Tahara [14]) were cultured in HUVEC Cells Medium (HCM): M199 + 0.25% Heparin + 20% FBS + 1% Penicillin/Streptomycin + 0.5% Endothelial Cells Growth Supplement (ECGS – Sigma Aldrich). HUVEC were co-cultured with cancer cells or stellate cells (activated or ATRA-treated) in organotypic 3D gels. Pancreatic cancer organotypic cultures were constructed as described previously [15], with some modifications introduced to successfully co-culture HUVEC. Briefly, 1 mL of solution constituted of 0.35 mL Type 1 Collagen, 0.35 mL Matrigel™, 0.2 mL HCM containing 5 × 10^5^ HUVEC and 0.1 mL FBS containing 5 × 10^5^ PSC was poured in a collagen pre-coated well of a 24-well plate. For triple cultures (HUVEC, PSC and PCC) cancer cells were added the following day suspended in the feeding medium.

Cell growth was monitored for the following seven days with dual-phase microscope. HUVEC survival, early sprouting and formation of vessel-like structures were monitored. Gels were harvested on day seven, fixed in formal-saline, embedded in paraffin and cut into 4 μm sections for H&E staining and immunostaining.

### 2.4 All trans retinoic acid (ATRA) treatment

ATRA (Sigma-Aldrich, Dorset, England) were dissolved in 100% ethanol and used as described before [9]. PSCs were rendered quiescent after treatment with 1 μM ATRA dissolved in medium for seven consecutive days as described before [9].

### 2.5 Immunostaining

Paraffin embedded organotypic sections were dewaxed and rehydrated. Heat induced epitope retrieval in citrate buffer (pH = 6) was used for all antibodies. Sections were permeabilized with 0.2% TritonX-100 and blocked with 2% bovine serum albumin (#K45-001; PAA laboratories), 0.02% fish skin gelatin (#G7765; Sigma), 10% FBS (#A15-104; PAA laboratories).

Primary antibodies were incubated at 4 °C overnight. Fluorescent-labelled appropriate secondary antibodies (Alexa fluor^®^ 488, 546) were incubated at room temperature for 1 h and nuclei were counterstained with DAPI. Controls were uniformly negative with appropriate isotype-specific immunoglobulin at matching dilution. Images were acquired and analysed using confocal microscopy (Carl Zeiss LSM 510).

### 2.6 Mice aortic rings angiogenesis assay

In order to study the angiogenic potential of the different cell lines the *ex vivo* mice aortic rings angiogenesis assay was performed as described before [16], with Institutional Review Board approval. Briefly, 12–16 weeks old wild type mice were sacrificed and aorta was surgically removed, cleaned, cut in rings (approximately 20 rings/aorta measuring 500 μm diameter) and incubated overnight in medium (OPTIMEM + Glutamax + 2.5% FBS, used throughout the experiment) at 37°, 5% CO_2_. The following day rings were embedded in solutions containing variable concentrations of Collagen (0.5 mg/ml, 1 mg/ml, 2 mg/ml and 4 mg/ml). On days one, four and seven the rings were fed with conditioned medium from AsPC1, Capan1, COLO357, activated or ATRA-treated PSCs with care being taken to ensure that the cell number is equal (ATRA-treated PSC proliferate slowly). To obtain conditioned medium from the above-mentioned cell lines, they were cultured in Opti-Mem + Glutamax + 2.5% FBS for 24 h (the same medium used as negative control). Supernatant was then centrifuged, filtered and immediately used to feed the rings (Supplementary Fig. 1).

Sprouting angiogenesis was quantified in all groups at days 6, 8 and 10 under dual-phase microscope. Rings were stained with *Bandeiraea simplicifolia (*BSI) Lectin-FITC (L9381 Sigma-Aldrich) and α-sma-CY3 (C619B Sigma-Aldrich) to obtain confocal images of the vascular structures as well as the supporting cells [16]. All experiments were run in triplicate.

### 2.7 Statistical analysis

Statistical analysis and graphical data representation were done using the software PRISM V.6 (Graphpad, La Jolla, USA). The normality of the distribution of the data was determined using D'Agostino & Pearson normality test. Summary data are expressed as the median with interquartile range (non-Gaussian distributions). Comparisons between different groups were performed using One-way ANOVA test with either Kruskal-Wallis or Friedman test for multiple comparisons, and Mann Whitney *U* test for two groups. Kaplan-Meier survival curves were generated for survival analyses using the Log Rank or Gehan-Breslow-Wilcoxon test. The level of significance was set at p < 0.05.

## 3 Results

### 3.1 Stroma specific hypovascularity of human PDAC

**Fig. 1:**
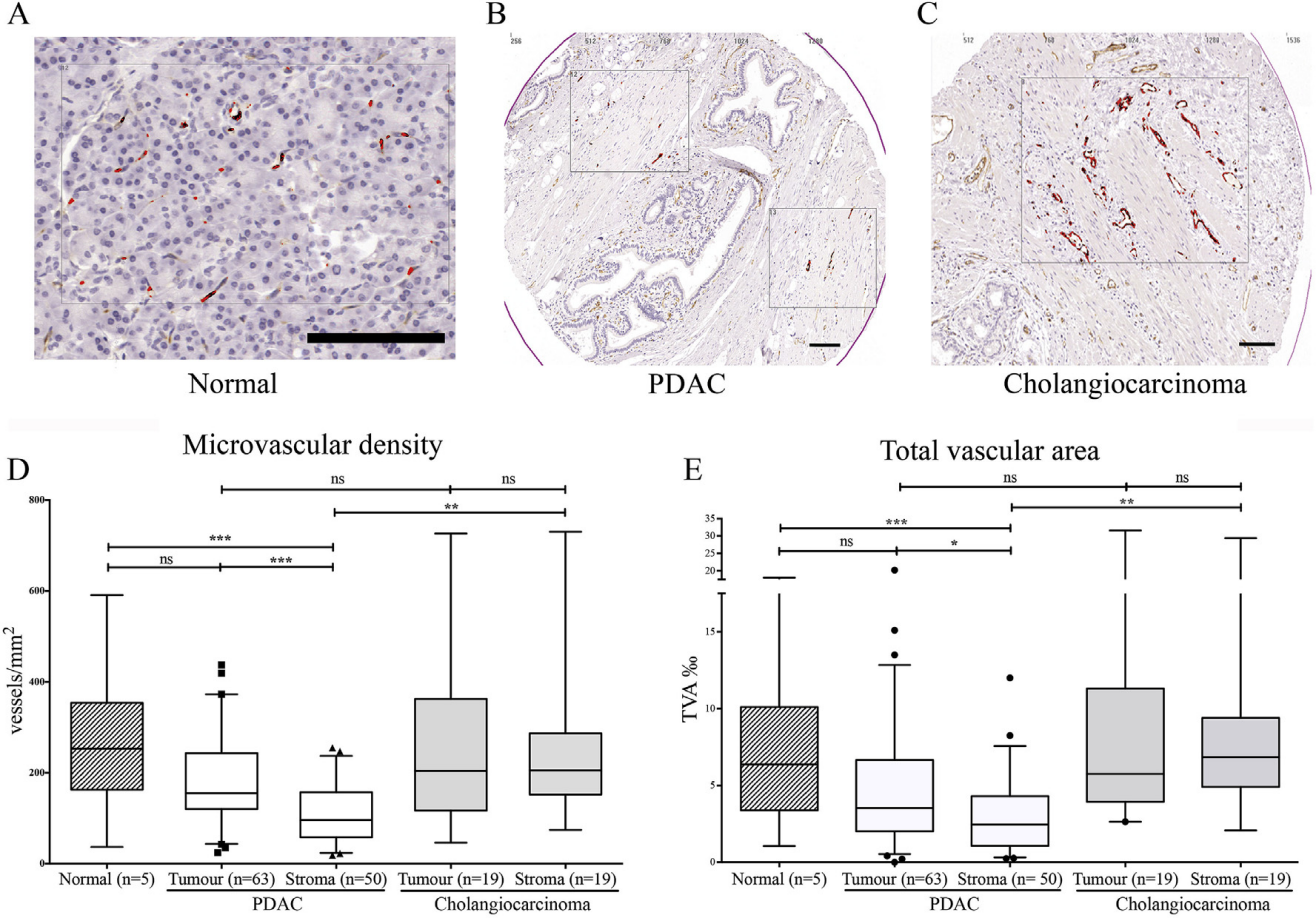
Stroma is hypovascular in PDAC but not cholangiocarcinoma. A–C: CD31 immuno-histochemistry (brown) was used to demonstrate vascular density in tissue micro-arrays with normal pancreas (A), PDAC (B) and cholangiocarcinoma (C). *Scale Bar* = *100 μm*. D-E The micro-vascular density (MVD, D) was measured by number of vessels/mm^2^. The total vascular area (TVA, E) was measured by area occupied by vessels per 1000 units of area using automated Ariol™ analysis for the tumour bearing and stromal areas of cancer. The summary data are shown in the form of box (median and interquartile ranges) and whisker (95% range) graphs. Each data-point represents a patient (numbers depicted by n=), which was derived as a median value of three to six microarray cores. Statistical analyses were performed by Kruskal-Wallis test with Dunn's post-hoc multiple comparison. ns, not significant, *p < 0.05, **P < 0.01, ***p < 0.001.

TMA Ariol™ analyses of normal pancreas, cholangiocarcinoma and PDAC tissues from 87 patients revealed significant differences in microvascular density (MVD), total vascular area (TVA), dimensions and distribution of vessels between normal and different cancer tissues (Fig. 1). Interestingly, the PDAC stromal compartment, but not cholangiocarcinoma stromal compartment, was significantly less vascular than either normal or tumoral tissue as measured by either MVD or TVA.

**Fig. 2:**
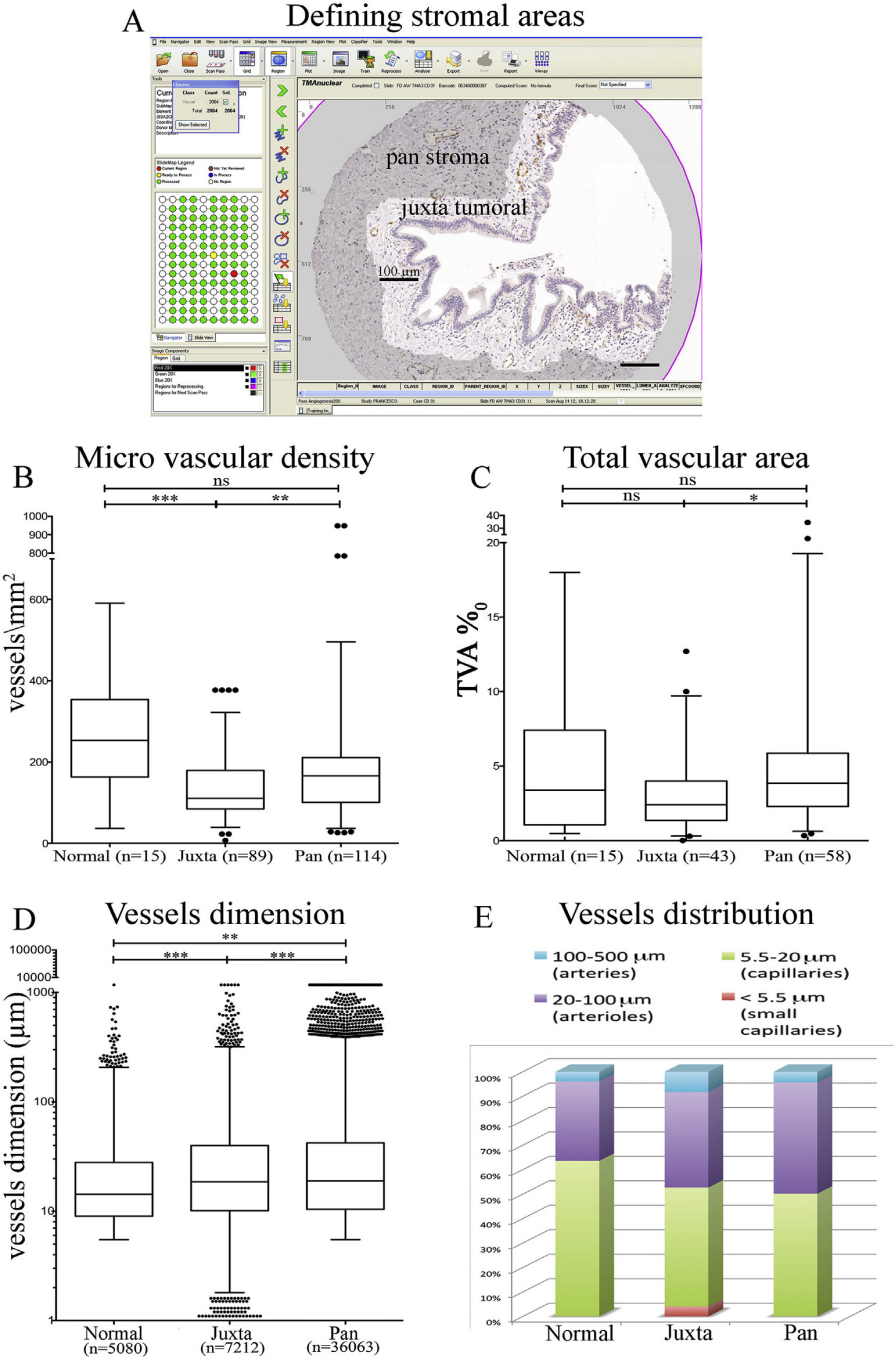
Vascular density of stromal sub-compartments. A: Juxtatumoral stroma (within 100 μm of tumour tissue) and panstroma (the rest of the tumour stroma) were defined using the Ariol™ software to perform separate analyses for vascular density. Scale Bar = 100 μm. B, C: The micro-vascular density (MVD, D) was measured by number of vessels/mm^2^. The total vascular area (TVA, E) was measured by area occupied by vessels per 1000 units of area using automated Ariol™ analysis for the stromal cub-compartments areas of PDAC. The summary data are shown in the form of box (median and interquartile ranges) and whisker (95% range) graphs. Each data-point represents a measurement from individual core. In case of multiple spots analysed within the same core (for Juxtatumoral only) the median of the index measurement per core was used. Statistical analyses were performed by Kruskal-Wallis test with Dunn's post-hoc multiple comparison. ns, not significant, *p < 0.05, **P < 0.01, ***p < 0.001. D: The dimension of each vessel was measured. The summary data are shown in the form of box (median and interquartile ranges) and whisker (95% range) graphs. Each data-point represents a vessel (number, n=) on a graph with logarithmic Y-axis scale. Statistical analysis was performed by Kruskal-Wallis test with Dunn's post-hoc multiple comparison. **P < 0.01, ***p < 0.001. E. The proportion of capillaries (5.5–20 μm) is the highest in normal tissue, whereas in tumour stroma arteries (100–500 μm) and arterioles (20–100 μm) are more prevalent. In juxta-tumoral stroma very small capillaries (<5 μm) account for 3–4% of total and may represent non-functional vessels compressed by the dense stroma.

Within the PDAC stroma, the juxtatumoral stroma (<100 μm of tumour, Fig. 2A) had significantly less vessel density (MVD) than either the panstromal compartment or normal pancreas. MVD for normal pancreas and panstromal compartment were similar (Fig. 2B). Surprisingly, there was no difference when comparing normal pancreas and juxtatumoral total vascular area (TVA) (Fig. 2C). We hypothesised that these subtle differences may be due to the differential distribution of vessels with different calibres. The dimensional spectrum of vessels in the healthy tissue was homogeneous and normally distributed as compared to tumoral stroma which had a skewed distribution (Fig. 2D). Whilst both stromal compartments demonstrate a heterogeneous vascular architecture, it is possible that in the juxta-tumoral stroma the microvessels are compressed by the dense stroma (Fig. 2E).

**Fig. 3:**
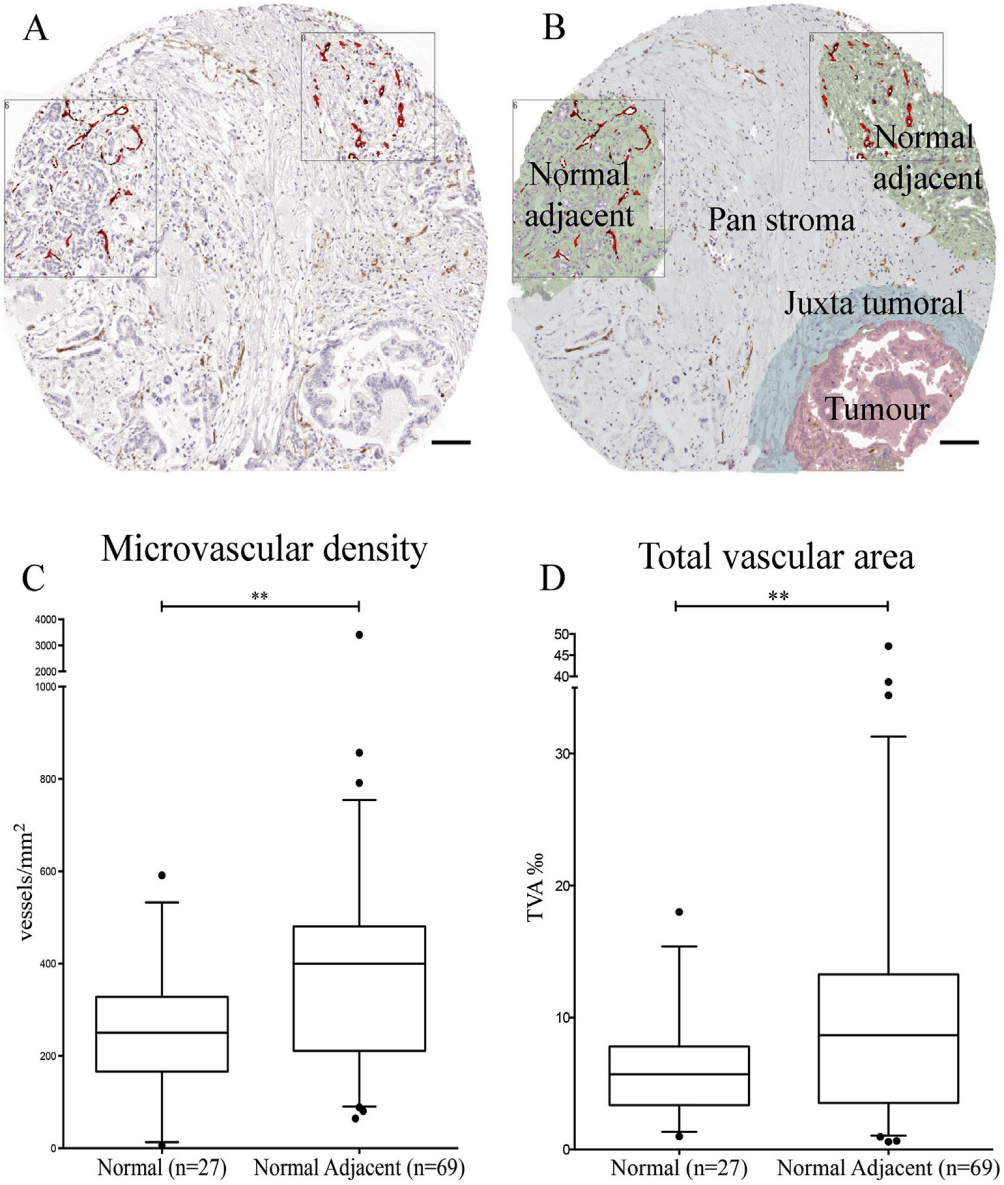
Normal adjacent pancreas is more vascularised in PDAC. A, B: Histopathological image of normal pancreas adjacent to PDAC showing higher vascular density, as identified by CD31 immuno-histochemistry. Scale Bar: 100 μm. C, D: The micro-vascular density (MVD, C) was measured by number of vessels/mm^2^. The total vascular area (TVA, D) was measured by area occupied by vessels per 1000 units of area using automated Ariol™ analysis for the normal adjacent areas in PDAC and normal pancreas. The summary data are shown in the form of box (median and interquartile ranges) and whisker (95% range) graphs. Each data-point is a selected area analysed. Statistical analysis by Mann-Whitney *U* test. **P < 0.01.

We subsequently analysed those areas, within samples coming from cancer patients, looking microscopically normal (preserved acinar structure) but in close proximity to the cancer surrounding stroma. Remarkably, this normal adjacent tissue was hyper-vascularised compared to all the tissue sub-compartments previously analysed (Fig. 3). Indeed, both micro vascular density (3C) and total vascular area (3D) of normal adjacent tissue resulted significantly higher than the correspondent values we had calculated in the tissue coming from healthy donors.

Thus, microscopic vascular distribution in PDAC does not resemble the symmetry of the cognate normal, functional organ or the heterogeneity seen on other cancers, affecting the same organ, such as cholangiocarcinoma. We postulated that these subtle stromal compartment differences may be because of the presence of activated pancreatic stellate cells (PSC) or immense extra-cellular matrix deposition in PDAC stroma.

### 3.2 Aortic ring angiogenesis assay

**Fig. 4:**
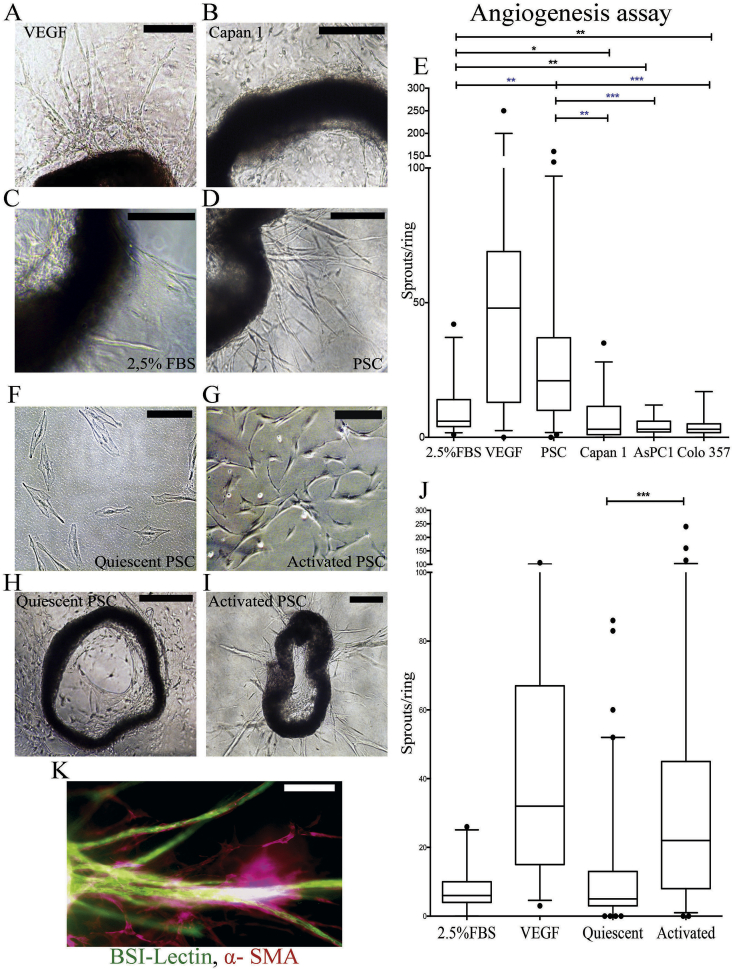
Stellate cells promote angiogenesis in mice aortic rings whereas cancer cells inhibit it. A–D: Representative microscopic images of aortic rings treated with either VEGF (30 ng/mL, positive control, A) [16], or conditioned medium from cancer cells (B), or negative controls (2.5% FBS supplemented media, C), or stellate cells (D) to demonstrate vessel sprouts at endpoint analysis. *Scale Bar* = *500 μm*. *E*: The summary data of the aortic ring angiogenesis assays are shown in the form of box (median and interquartile ranges) and whisker (95% range) graphs. Each data-point represents an aortic ring with experiments performed in multiple replicates [6], [7], [8], [9], [10], [11], [12] with at least three biological replicates. Statistical analyses were performed by Kruskal-Wallis test with Dunn's post-hoc multiple comparison. *p < 0.05, **P < 0.01, ***p < 0.001. F, G: ATRA treated pancreatic stellate cells show a quiescent morphology, whilst ethanol treated pancreatic stellate cells show the characteristic myofibroblast-like morphology. Scale Bar = 50 μm. H,I: Representative microscopic images of aortic rings treated conditioned medium from ATRA-treated PSC (H) or activated PSC (I) at endpoint analysis. *Scale Bar* = *500 μm (H) and 200 μm (I)*. J: The summary data of the aortic ring angiogenesis assays are shown in the form of box (median and interquartile ranges) and whisker (95% range) graphs. Each data-point represents an aortic ring with experiments performed in multiple replicates [6], [7], [8], [9], [10], [11], [12] with at least three biological replicates. Statistical analyses were performed by Kruskal-Wallis test with Dunn's post-hoc multiple comparison. ***p < 0.001. K: Confocal microscope image of a sprout confirming the vascular nature of the sprouts. Vascular structures are stained with BSI-Lectin and supporting fibroblasts (pericytes) with smooth muscle actin (SMA). *Scale bar*: *100 μm*. See Supplementary Fig. 2 for other analysis on effect of Collagen concentration.

*Ex vivo* angiogenesis assays using mice aortic rings experiments demonstrated a pro-angiogenic feature of activated pancreatic stellate cells (aPSC) and significant anti-angiogenic influence exerted by cancer cells (Fig. 4A–E). Rendering PSC quiescent with ATRA [9] reversed this pro-angiogenic effect (Fig. 4H–J). Immunofluorescent staining of the rings confirmed the vascular nature of the sprouts (Fig. 4K): endothelial cells were BSI-Lectin positive, support cells and pericytes were αSMA-positive [16].

Progressively increasing concentrations of collagen within the embedding gel influences the formation of sprouts in a bimodal effect, intermediate concentrations being the most suitable for angiogenesis, as reported in the past [17], [18] (Supplementary Fig. 2).

### 3.3 3D organotypic cultures

**Fig. 5:**
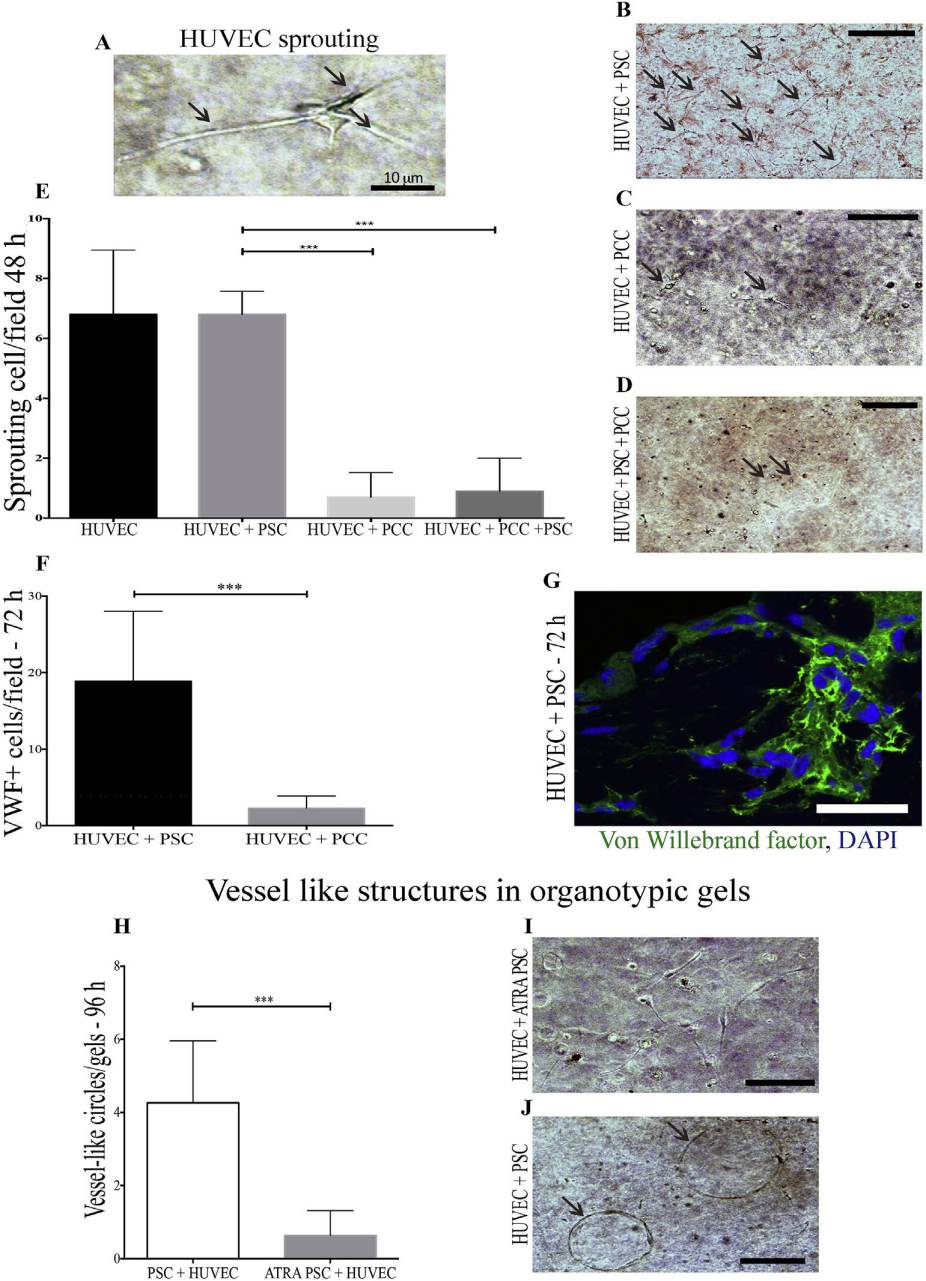
Activated PSC promote HUVEC survival and angiogenesis in organotypic cultures. A: Early sprouting (24 h) of a single HUVEC cell in a 3D organotypic culture. B–D: Representative images of sprouting HUVEC cells (arrows) in 3D double co-culture with PSC (B) or double culture with PCC (C) or triple HUVEC/PSC/PCC cultures (D) at 48 h after starting the cultures. E: Summary data are mean + SEM of sprouting HUVEC cells per field analysed from multiple organotypic cultures as shown in B–D. 6 OT gels were cultured of each condition. 10 independent fields were analysed for each double culture. Statistical analyses were performed by Friedman Test, *p < 0.05, **P < 0.01, ***p < 0.001). F, G; Von-Willebrand Factor positive HUVEC (G) survived significantly more in OT gels with HUVEC-PSC than HUVEC-PCC co-cultures. Summary data are mean + SEM of sprouting cells per field analysed. 15 independent fields were analysed for each slide stained from a total of eight biological samples. Statistical analyses were performed by Friedman Test, ***p < 0.001. H–J: Circular structures resembling vessels were detectable after 72–96 h in organotypic gels with activated PSC-HUVEC (I) significantly more than in gels with ATRA-treated PSC-HUVEC (J). 12 double culture gels were analysed. Summary data are mean + SEM of circular structures per field analysed. Statistical analyses were performed by Friedman Test, ***p < 0.001.

HUVEC cells, in the appropriate collagen/Matrigel 3D mixture demonstrated the ability of early sprouting (Fig. 5A) at 48 h and aggregating to form luminal structures at 72 h 3D organotypic co-cultures demonstrated strong anti-angiogenic signalling from pancreatic cancer cells (PCC). Indeed, the presence of PCC inhibited endothelial cells survival both in the ‘double’ HUVEC-PCC and the ‘triple’ HUVEC-PCC-PSC organotypic cultures (Fig. 5E). There was almost no endothelial cells detectable 48–72 h after the co-culture started with any of cancer cell lines tested (Fig. 5 C and D). On the other hand, HUVEC were not only detectable, but also sprouting (Fig. 5B) and assembling in luminal structures (Fig. 5J) in the presence of aPSC. Von-Willebrand factor immuno-fluorescent staining confirmed the vascular nature of the circular vessel like aggregations of HUVEC in 3D (Fig. 5G). The number of endothelial cells (von Willebrand factor positive) present 72 h after 3D double cultures was significantly lower when HUVEC were co-cultured with PCC than with PSC (Fig. 5F). ATRA-induced stellate cells quiescence suppressed this pro-angiogenic activity, together with the previously shown capacity to shrink the organotypic gel (Fig. 5H and I). Of note, a higher Collagen concentration in the gels [2] suppressed HUVEC survival (data not shown).

Taken together, these data draw the picture of a fibrovascular gradient, in which cancer cells signalling as well as dense ECM contribute to render the juxtatumoural the less vascular compartment, while activated stellate cells promote angiogenesis towards the edges of the tumour mass.

### 3.4 Impact on patient survival

Patient data are summarised in Supplementary Table 1. Three patients experiencing postoperative mortality (<30 days) were excluded from survival analysis. Briefly, PDAC patients (n = 63) had worse prognosis than those with cholangiocarcinoma (n = 19) (median = 403 vs 544 days, *p* = *0.04*). In PDAC patients, sex and lymph involvement did not correlate directly with the prognosis, whereas the well-differentiated grade was associated with a better outcome (Supplementary Fig. 3).

**Fig. 6:**
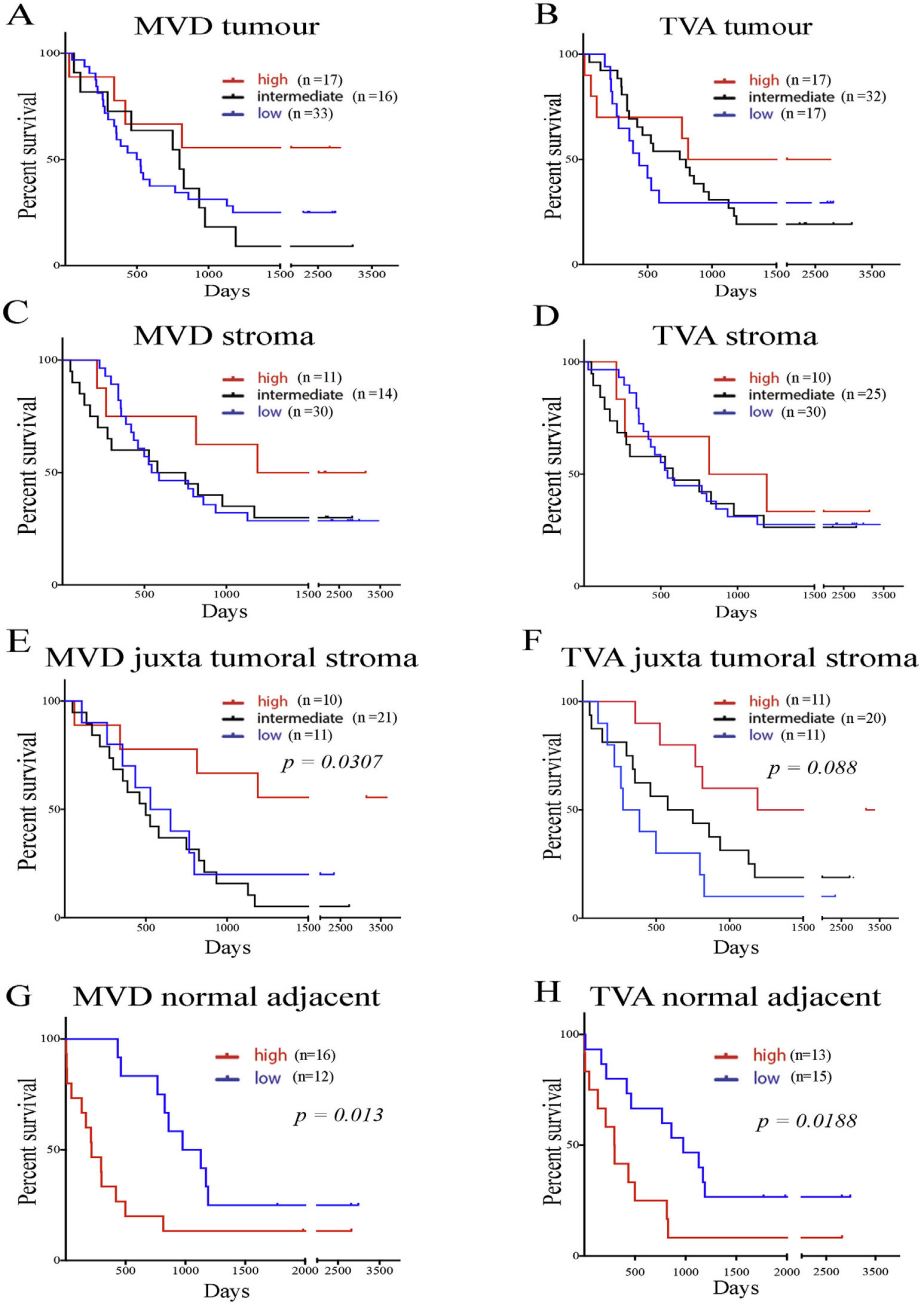
Juxtatumoral and normal adjacent vascular density impact on prognosis. Kaplan Meier curves were obtained after patients were divided either in three groups based on quantiles which were high (above 3rd quartile), intermediate (between 1st and 3rd quartile), or low (below 1st quartile) for total vascular area (TVA) and microvascular density (MVD). Comparisons were made by Log-rank (Mantel Cox) test. For each patient (number, n=), data are obtained by calculating the average MVD and TVA measured within the specific parenchymal area for that patient. A–D: Whole tumoral (A,B) and stromal (C,D) vascular density indices do not have prognostic impact, E–F: Higher juxtatumoral vascular density indices for MVD (E) and TVA (F) result in better prognosis. G,H: For normal adjacent pancreas the data were analysed in two groups as high (above median) or low (below median). Both vascular indices MVD (G) and TVA (H) in tumour adjacent normal tissues indicate higher vascularisation leads to poorer prognosis. See Supplementary Fig. 3 for other analysis on cholangiocarcinoma.

The prognostic value of the vascular indices TVA and MVD was analysed within different areas of the neoplastic pancreas. Neither the whole tumoral nor the whole stromal compartment vascularisation showed any statistical correlation with the outcome (Fig. 6A–D). Two specific stromal compartments' (juxta-tumoral stroma and normal adjacent tissue) vascular density (both MVD and TVA) impacted patient's prognosis. Interestingly, the increased vascularisation of the juxtatumoral compartment was associated with prolonged survival, whereas both enhanced MVD and TVA within the normal adjacent tissue was associated with worse prognosis (Fig. 6E–H).

These findings would confirm the proposed model of development of PDAC foci, based on a fibrovascular gradient. For example, the most aggressive cancer exhibited the highest gradient i.e., having the less vascular juxtatumoral compartment and the most vascular normal adjacent tissue.

## 4 Discussion

In this report, we demonstrate that the stromal sub-compartment specific vascularisation is different, and is clinically relevant in human PDAC. Radiologically it has been noted that intra-tumoral perfusion indices progressively increase from the core to the outer rim of PDAC [17]. We suggest the activated pancreatic stellate cells (PSC) may play a role in this differential vascular density. We have previously demonstrated that increasing the PSC proportion drives aggressive cancer behaviour, reiterating the concept that cancer development is dynamic: cellular behaviour as well as ECM composition are subject to spatio-temporal variations [1], [32]. This, in turn, can dictate behaviour of other critical stromal cell populations such as immune cells. For example, CD8^+^ cytotoxic T cells could not infiltrate juxta-tumoral sub-compartment [7].

PSC's impact on angiogenesis possibly is dependent on their activation status via modulation of secretome and the subsequent ECM composition. PSC have been shown to secrete a number of pro-angiogenic factors such as vascular endothelial growth factor (VEGF) and Periostin among others in a hypoxia-dependent manner. Activated PSC produce excessive amount of ECM proteins, mainly Collagen and Fibronectin, contributing to dense stroma which is anti-angiogenic [18], [19], [20]. In our models, activated PSC stimulates endothelial cell growth in 3D ‘double’ cultures and PSC supernatant induces aortic rings sprouting. We have also shown that ATRA-induced PSC quiescence suppresses the PSC angiogenic potential. Of note, the presence of cancer cells in ‘triple’ 3D organotypic influences endothelial cells adversely, but does not result in increases apoptosis (Supplementary Fig. 4), which in conjunction with results from aortic rings demonstrate the anti-angiogenic profile of PCC. Previously we had demonstrated that 3D organotypic cultures of PSC and PCC, PSC activation by PCC can alter the gel composition rendering them stiffer and contracted. This effect might be mediated by secretion of collagen of fibronectin from activated PSC or enhanced cross-linkage of existing Collagen [1], [20]. Thus, eliciting the individual direct and indirect contribution of PSC, PCC and ECM proteins, to name just the main players, upon endothelial cell behaviour in the complex PDAC micro-environment remains difficult when all constituents are admixed. Certainly targeting PSC does seem to alter the vascular density *in vivo* [33].

Collagen has previously been demonstrated to be anti-angiogenic in a number of assays [21], [22]. In our organotypic model, increasing relative concentration of collagen in the gel adversely affected endothelial cells' survival. Similarly, increasing the Collagen proportion in the embedding aortic rings gel, we observed a significant reduction in sprouts. Thus, rather than an overall biological pro-angiogenic effect from the PSC secretome as demonstrated by the aortic ring angiogenesis assays, PSC may play a dynamic influence on pancreatic cancer vascularisation, by modulating the ECM to spatio-temporally to influence the vascular network.

In our experimental models, PCC (AsPC1, Colo357 and Capan1) exerted a strong anti-angiogenic effect, inhibiting HUVEC survival in 3D co-cultures as well as sprouting angiogenesis of mice aortic rings. A similar effect was shown in 2D cultures by Erkan et al. and was attributed to the PCC secretion of Endostatin, a strong anti-angiogenic agent derived from Collagen degradation [23]. Paracrine Endostatin secretion effect from PCC may explain the selective hypovascular nature of juxta-tumoral stroma.

However there are other possible molecular explanations for the differential vascular distribution, and indeed all of them may contribute in a spatio-temporal manner. Periostin, is expressed almost exclusively in stroma and is undetectable in healthy pancreatic [24]. Activated PSC secrete Periostin in PDAC [24], [25] and stimulate an auto-activation loop for PSC, promote cancer cells survival under hypoxic conditions; thus create a tumour-supportive microenvironment [26]. Moreover, Periostin has been shown to promote angiogenesis, and slow down the ECM turnover inhibiting a number of matrix metalloproteinases (MMP) and promoting the activity of tissue inhibitors of metalloproteinases (TIMP) [26], placing Periostin to be an active player in tissue remodelling. Notably, the deposition of Periostin from activated PSC selectively happens towards the invasive front of the cancer [24] and this may influence the angiogenic activity.

Similarly, L1CAM (Cell Adhesion Molecule L1) is deposited on the invasive front of PDAC [27]. L1CAM recruits of endothelial cells from the expanding tumour, as well as the invasiveness of cancer cells [28]. These are possibly some of the molecular mechanisms which could explain the gradient of vascularisation observed by us.

From the analysis of our *in vitro* models model we propose, that the interplay remains complex and no single factor is dominant at all times in the spatio-temporal distribution of vessels in human PDAC. Indeed, depleting stroma in murine PDAC models, via either selective deletion of αSMA positive fibroblasts or Sonic Hedgehog (SHH) system in cancer cells, resulted in less differentiated, more vascularised and more aggressive tumours with a worsening overall survival in murine models [5], [6]. Hence, modulating, rather than ablating PDAC stroma may be more useful strategy [8]. Current attempts to target newly formed tumoral vessels functional stabilization pathways (i.e. Notch/DLL or Semaphorins) could conceptually be more successful in view of our findings [29], [30], [31]. Further studies are needed in order to identify selective markers of the invasive front and the hypoxic core which may further mechanistically implicate the differential vascular gradient.

## Conflicts of interest and disclosures

All authors have nothing to disclose.

## Figures and Tables

**Figure:**
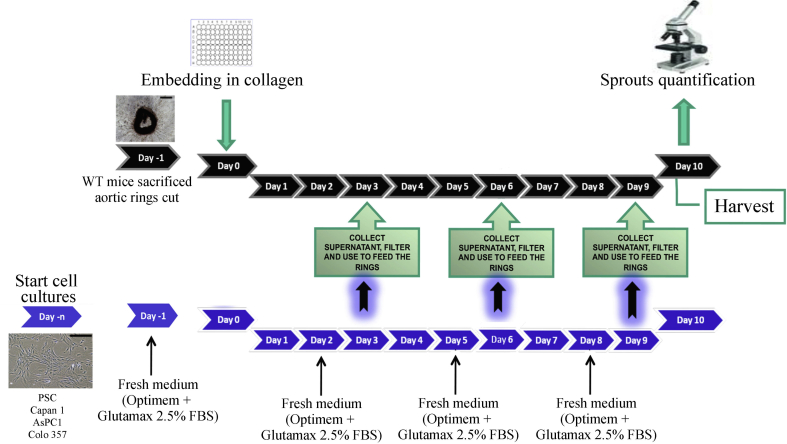
Supplementary Fig. 1Aortic rings angiogenesis assay. Schema of construction of aortic ring angiogenesis assays and feeding the aortic rings with conditioned media from cancer or stellate cells.

**Figure:**
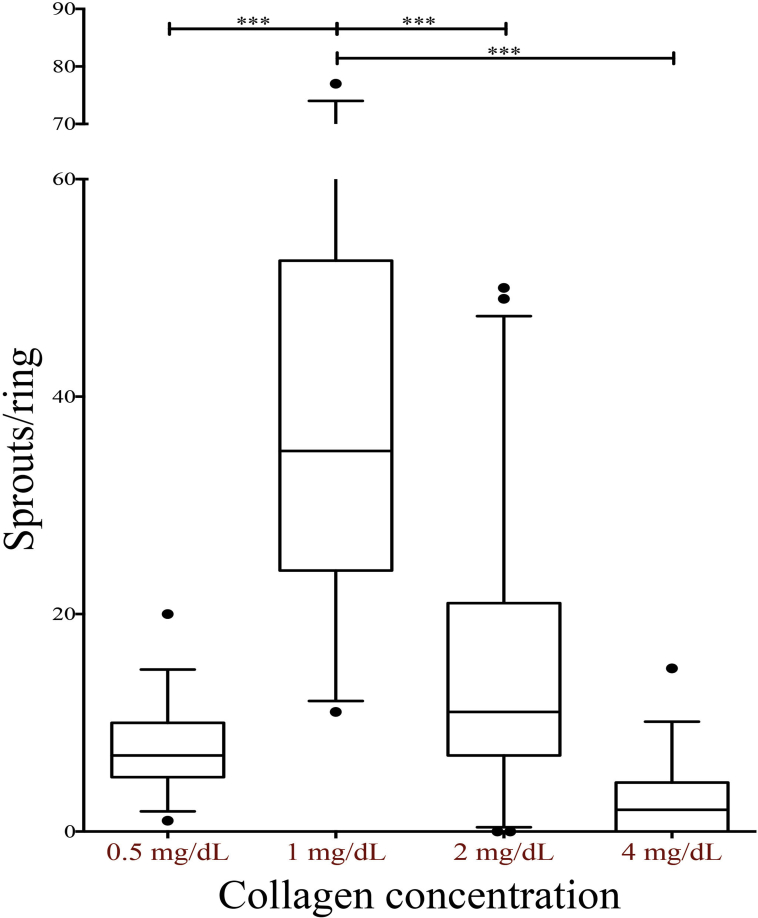
Supplementary Fig. 2Effect of collagen concentration on angiogenesis in mice aortic rings. The summary data of the aortic ring angiogenesis assays are shown in the form of box (median and interquartile ranges) and whisker (95% range) graphs. Each data-point represents an aortic ring with experiments performed in multiple replicates [6], [7], [8], [9], [10], [11], [12] with at least three biological replicates using different Collagen concentrations to embed the aortic rings. Statistical analyses were performed by Kruskal-Wallis test with Dunn's post-hoc multiple comparison. *p < 0.05, **P < 0.01, ***p < 0.001.

**Figure:**
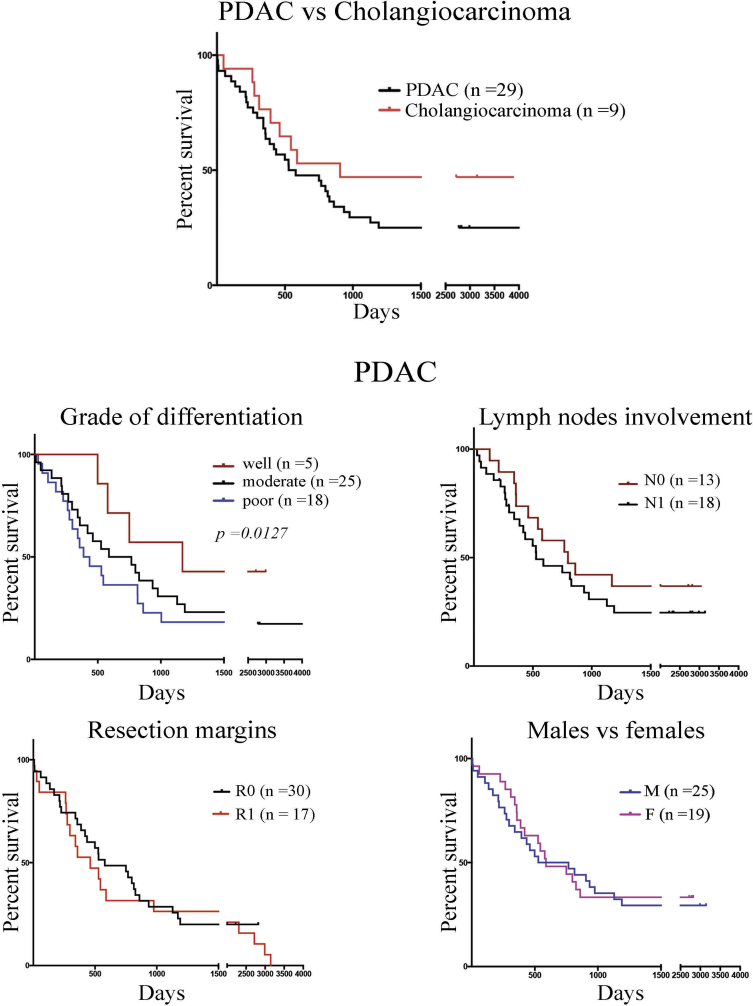
Supplementary Fig. 3Juxtatumoral and normal adjacent vascular density impact on prognosis. Kaplan-Meier curves were obtained after patients for various factors which may have prognostic impact. Comparisons were made by Log-rank (Mantel Cox) test. For each patient (number, n=), data are obtained by calculating the average MVD and TVA measured within the specific parenchymal area for that patient. A: PDAC versus cholangiocarcinoma overall survival in this patient cohort, B–E: Grade of tumour (B), lymph nodal involvement (B), resection margin involvement and impact on sex in patients with PDAC and their survival.

**Figure:**
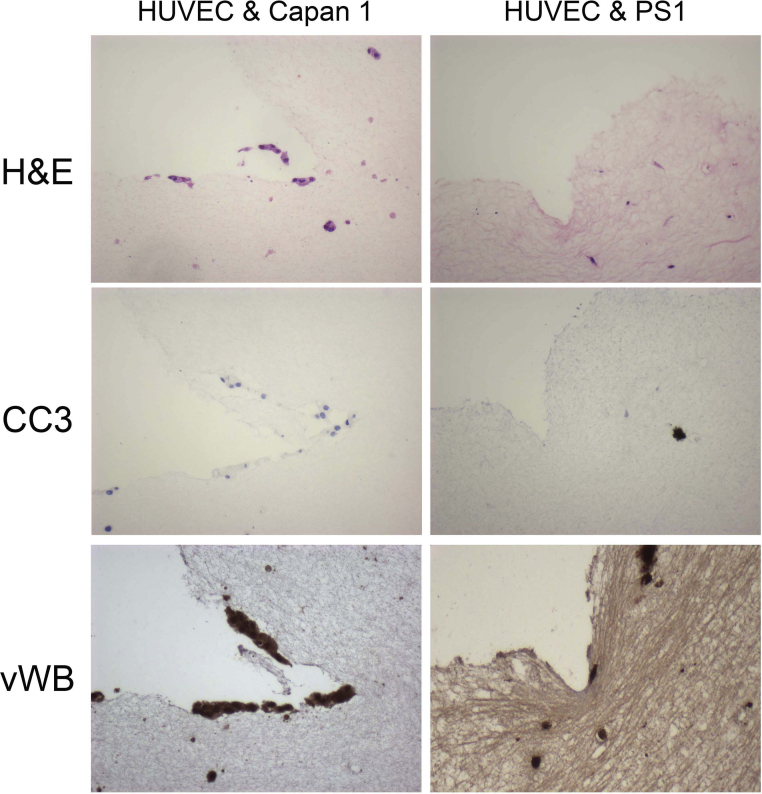
Supplementary Figure 4Organotypic cultures' consecutive sections stained for cleaved caspase 3 and von Willebrand factor. HUVEC-Capan1 and HUVEC-PSC double organotypic cultures were used. Consecutive sections were stained for stained for cleaved caspase 3 and von Willebrand factor.
