# Supplementary material for: Pancreatic stellate cells regulate blood vessel density in the stroma of pancreatic ductal adenocarcinoma
Source: Pancreatology. 2016 Nov-Dec;16(6):995–1004. doi: 10.1016/j.pan.2016.05.393 (PMC5123629; doi:10.1016/j.pan.2016.05.393)
Supplement: Supplementary file 1 [file mmc1.docx]

**Supplementary Table 1: Patient characteristics**

| Characteristic | Attribute | Distribution |
| --- | --- | --- |
| Diagnosis | PDAC | 63 |
|  | Cholangiocarcinoma | 19 |
|  | Healthy | 5 |
| Age | Median (IQR) | 72 (64-79) |
| Sex | M:F | 47:40 |
| Tumour stage | T1 | 10 |
|  | T2 | 25 |
|  | T3 | 45 |
| Nodal stage | N0: N1 | 28:48 |
| Grade | Well | 8 |
|  | Moderate | 35 |
|  | Poor | 31 |

**Supplementary Table 2: Antibodies used for assays.**

| **Antibody** | **Mehtod** | **Catalogue ID, Company** |
| --- | --- | --- |
| CD31 | IHC | Ab28364 ABCAM |
| α-Smooth muscle actin | IF | C6198 SIGMA |
| Von Willebrand Factor | IF | F3520 SIGMA |
| BSI Lectin-FITC | IF | L9381 SIGMA |
| Goat anti-mouse 546 | IF, secondary | A11030 INVITROGEN |
| Goat anti-rabbit 488 | IF, secondary | A11070 INVITROGEN |
